# A new tool to sense pH changes at the neuromuscular junction synaptic cleft

**DOI:** 10.1038/s41598-020-77154-3

**Published:** 2020-11-24

**Authors:** Matías Blaustein, Sonia Wirth, Gustavo Saldaña, Ana Paula Piantanida, María Eugenia Bogetti, María Eugenia Martin, Alejandro Colman-Lerner, Osvaldo D. Uchitel

**Affiliations:** 1 Departamento de Fisiología, Biología Molecular y Celular, Facultad de Ciencias Exactas y Naturales (FCEN), Universidad de Buenos Aires (UBA), Buenos Aires, Argentina; 2 Instituto de Biociencias, Biotecnología y Biología Traslacional (iB3), FCEN, UBA, C1428EHA Buenos Aires, Argentina; 3 Instituto de Biodiversidad y Biología Experimental y Aplicada (IBBEA), FCEN, CONICET-UBA, Buenos Aires, Argentina; 4 Instituto de Fisiología, Biología Molecular y Neurociencias (IFIBYNE), CONICET-UBA, C1428EHA Buenos Aires, Argentina; 5 Instituto de Biología Celular y Neurociencias (IBCN) Dr. Eduardo de Robertis, Facultad de Medicina, CONICET-UBA, Buenos Aires, Argentina

**Keywords:** Molecular neuroscience, Synaptic transmission, Microscopy, Biosensors

## Abstract

Synaptic transmission triggers transient acidification of the synaptic cleft. Recently, it has been shown that pH affects the opening of postsynaptic channels and therefore the production of tools that allow to study these behaviors should result of paramount value. We fused α-bungarotoxin, a neurotoxin derived from the snake *Bungarus multicinctus* that binds irreversibly to the acetylcholine receptor extracellular domain, to the pH sensitive GFP Super Ecliptic pHluorin, and efficiently expressed it in *Pichia pastoris*. This sensor allows synaptic changes in pH to be measured without the need of incorporating transgenes into animal cells. Here, we show that incubation of the mouse levator auris muscle with a solution containing this recombinant protein is enough to fluorescently label the endplate post synaptic membrane. Furthermore, we could physiologically alter and measure post synaptic pH by evaluating changes in the fluorescent signal of pHluorin molecules bound to acetylcholine receptors. In fact, using this tool we were able to detect a drop in 0.01 to 0.05 pH units in the vicinity of the acetylcholine receptors following vesicle exocytosis triggered by nerve electrical stimulation. Further experiments will allow to learn the precise changes in pH during and after synaptic activation.

## Introduction

The content of synaptic vesicles has been shown to be acid^1^, depending on a proton ATPase that causes the electrochemical gradient necessary to transport neurotransmitters into the vesicle^2^. During synaptic transmission, the synaptic vesicle fuses to the presynaptic membrane and its content is released into the synaptic cleft. During this event, vesicular pH rises and falls again after endocytosis^1^, which is consistent with proton release into the synaptic cleft. Taking into account the very small volume of a synaptic vesicle (2 × 10 ^−20^ L) and the estimated pH before exocytosis (around 5.5), it has been calculated that there is less than one free proton per vesicle^3^. However, upon vesicle opening a process which includes deprotonation of proteins and neurotransmitters takes place and over 50 protons are released per vesicle^3^. Therefore, the synaptic cleft, which is a space 50 nM wide, is exposed to changes in pH due to the release of vesicle content and also to transmembrane fluxes owing to Ca^2+^/H^+^ exchange by the plasma membrane Ca^2+^ ATPase^4^. A transient drop in extracellular pH has been reported during synaptic activity in hippocampal sections^5^. It has been proposed that in photoreceptor terminals the liberation of vesicular protons results in an acidification of the synaptic cleft from 7.5 to 6.9, depending on the feedback inhibition of Ca^2+^ channels^6^. This study also showed that pH oscillations depend on the concentration of protons in the extracellular solution and that acidification is insignificant in acutely dissociated terminals, demonstrating that an intact synaptic cleft is essential.

Recently, Super Ecliptic pHluorin (SEP)^1^, a pH-sensitive green fluorescent protein, was fused to the extracellular domain of a postsynaptic membrane protein and used in lateral amygdala pyramidal neurons to report changes in extracellular pH at dendrite synapses^7^. After stimulation, the transfected pyramidal neurons transiently acidified neighboring dendrites and spines, a phenomenon which was followed by a slower increase in pH. The extent of pH fluctuations depended on stimulus frequency although quantification of the changes in proton concentration was not reported. In contrast to the established dogma, Stawarski et al. recently used genetically encoded fluorescent pH indicators to demonstrate that glutamatergic synaptic clefts alkalinize rather than acidify during neurotransmission at both the mouse calyx of Held and *Drosophila* neuromuscular junction (NMJ)^8^. Therefore, the exact changes in pH during synaptic transmission are not fully resolved.

Changes in pH at the synaptic cleft at the mammalian NMJ have not been documented. With this aim, we developed a probe by fusing α bungarotoxin (BTX), a neurotoxin derived from the snake *Bungarus multicinctus* that binds irreversibly to the acetylcholine receptor extracellular domain, to SEP^1^ and efficiently expressed this fusion protein in *Pichia pastoris*. Using this tool, we were able to detect a drop in pH in the vicinity of the acetylcholine receptors following vesicle exocytosis triggered by nerve electrical stimulation.

## Results

**Figure 1:**
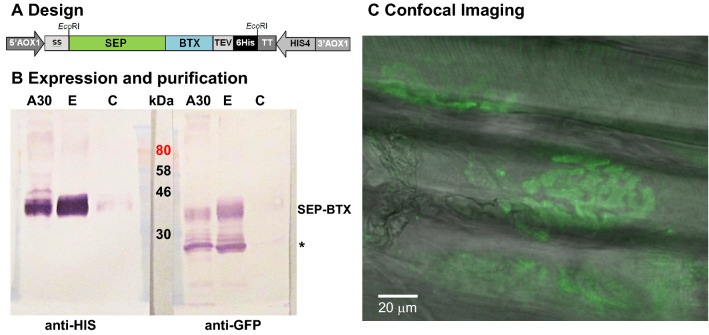
**A** Construction design for the expression of SEP-BTX in *P. pastoris*. The DNA coding sequence for the SEP-BTX fusion protein was inserted into the *Eco*RI site of the expression vector pPIC9. It was cloned downstream and in frame with the alpha factor signal sequence (ss) of *S. cerevisiae* and upstream of the coding sequences for the recognition peptide for tobacco etch virus protease (TEV) and a 6 × His tag (6His). 5′AOX1, TT and 3′AOX1: promoter region, transcription terminator and 3′ region of *P. pastoris* alcohol Oxidase 1 gene, respectively. HIS4: Histidinol dehydrogenase gene for selection of transformants in minimal medium. **B** Western blot of recombinant SEP-BTX purification fractions revealed with anti-His tag (left) or anti-GFP (right) antibodies. Culture supernatant (C) was concentrated by ultrafiltration with a 30 kDa Amicon Ultra (A30) and SEP-BTX was purified and eluted (E) by gravity flow Ni–NTA affinity chromatography. Marker: Colorplus Prestained Protein Marker (New England Biolabs). The expected molecular weight of the fusion protein is around 37 kDa. A smaller fragment was also detected by the anti-GFP antibody (*). The presence of this fragment did not prevent proper binding of SEP-BTX to the acetylcholine receptor. **C** Confocal image of a levator auris NMJ junction incubated with the SEP-BTX fusion protein and captured with Nomarsky optics (488 nM wavelength light excitation, 510 nM emission). Fluorescence and transmission images were merged.

**Figure 2:**
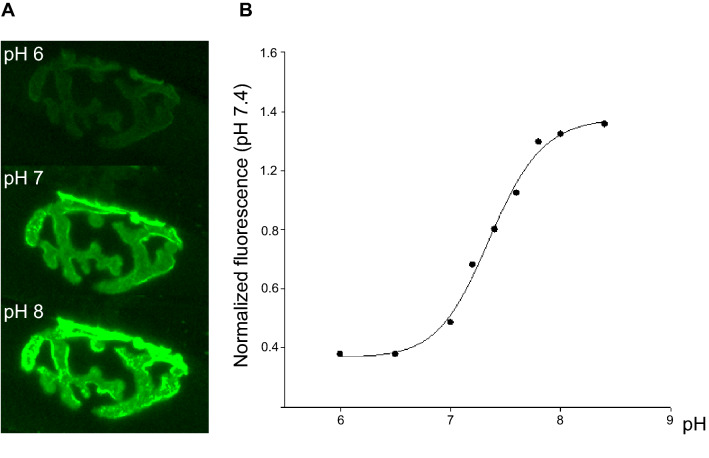
**A** The fluorescence image shows the NMJ incubated with SEP-BTX fusion protein bathed with 10 mM HEPES saline buffered at pH 6, 7, and 8. **B** Calibration curve (average of n = 3) obtained by measuring the fluorescence intensity of the NMJ incubated at different pHs. The value at pH 6 and 9 were considered the minimal and maximal fluorescence outputs, respectively (for calibration curve, see main text).

SEP-BTX was cloned in pPic9, expressed in *P. pastoris* and purified, resulting in an active protein of the expected size for de fusion (37 kDa, Fig. 1A,B). Muscles incubated with SEP-BTX washed and mounted in the recording chamber were screened looking for NMJ´s following the thin nerve branches with Nomarsky interference optics. NMJ´s were illuminated with a 488 nM wavelength light source to excite the SEP-BTX fusion protein attached to the postsynaptic acetylcholine receptor. Typical mouse NMJ pretzel like images as those displayed in Figs. 1C and 2A were observed.

The sensitivity to pH of the SEP-BTX fusion protein probe was investigated by measuring the fluorescence intensity of the NMJ incubated at different pHs in a high capacity buffer (HEPES buffer 10 mM). A fluorescence vs pH curve was constructed taken the value at pH 9 as the maximal fluorescent output (Fig. 2A,B) and the following equation *Y* = *y*_0_ + *y*_max_/(1 + 10^(pK-pH)^) was fitted to the data, where *y*_0_ represents the offset, *y*_max_ the dynamic range, and pK the pH where fluorescence is half maximal, a value that corresponds to minus the logarithm of the equilibrium constant for protonation^9^. The pK from the fit was 7.36.

**Figure 3:**
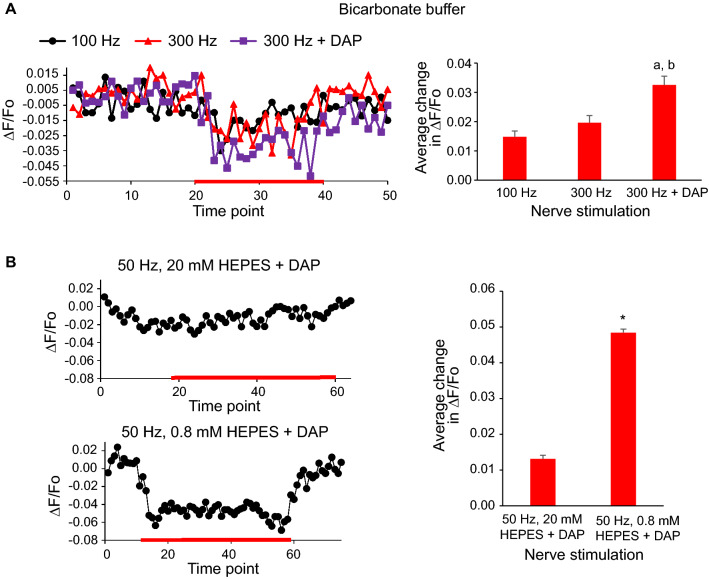
**A** Left: examples of NMJ fluorescence intensity changes before, during and after nerve stimulation at 100, 300 Hz and 300 Hz plus 100 µM DAP. The red line indicates the interval during which the nerve was stimulated. Right: the average changes in NMJ fluorescence intensity were calculated for each type of nerve stimulation. Letters indicate significant differences (Student’s t-test): a. *p* < 0.01 compared to stimulation at 100 Hz. b. *p* < 0.01 compared to stimulation at 300 Hz. **B** Left: comparison of NMJ fluorescence intensity changes during nerve stimulation at 50 Hz at the indicated HEPES buffer concentrations in the presence of 100 µM DAP. Right: the average changes in NMJ fluorescence intensity were calculated for each type of nerve stimulation. * *p* < 0.01 compared to stimulation in 20 mM HEPES.

The effect of nerve stimulation on the NMJ fluorescence was studied measuring the fluorescence intensity before, during and after nerve stimulation at 50, 100 or 300 Hz. As shown in Fig. 3A, a drop in the fluorescence was recorded during stimulation at 100 and 300 Hz in a bicarbonate buffer solution. The maximum pH change was estimated based on the calibration curve to be of 0.024 + / − 0.006 (n:4) for the 300 Hz stimulation and less than 0.02 pH unit for the 100 Hz stimulation. In another series of experiments the synaptic output was increased by incubating the nerve muscle preparation with the K^+^ channel blocker diaminopiridine (DAP, 100 µM, Sigma Aldrich, USA), known to increase the amplitude and prolong the time course of transmitter release^10^. As a result of DAP addition, fluorescence changes were significatively stronger (Fig. 3A).

To demonstrate that the observed changes in fluorescence were due to a change in pH in the synaptic cleft, the buffer capacity of the bathing solution was increased by replacing the bicarbonate buffer with a HEPES 20 mM pH 7.3 solution in the presence of DAP. In these experimental conditions, fluorescence changes due to nerve stimulation were not observed (Fig. 3B). In contrast, when the muscle was incubated with a lower HEPES buffer concentration (0.8 mM), the stimulation of the nerve induced a clear reduction in fluorescence, even at a 50 Hz stimulation, which corresponded to a drop in pH of 0.032 + / − 0.008 (Fig. 3B, n:3).

## Discussion

The experiments reported here have demonstrated that it is feasible to measure pH changes at the synaptic cleft using a fusion between a compound which binds to the postsynaptic receptor with high affinity and a pH-sensitive probe ready to capture the protons released into the synaptic cleft. This sensor allows synaptic changes in pH to be measured obviating the need of incorporating transgenes into animal cells, a process that can potentially lead to perturbations associated with their exogenous expression.

Our experimental results using SEP-BTX have shown that acidification takes place at the NMJ synaptic cleft, confirming previous reports in other synapses^3,11–16^. A drop in pH during synaptic activity at the NMJ was suggested to be associated to the modulatory effect of the Acid Sensing Ion Channels (ASIC) on neuromuscular transmission^17^. Activation of ASICs requires changes in pH of 0.5 units or more, a value not reached in our measurements. One possibility is that the sensor also binds to other acetylcholine receptors located outside the synaptic cleft. Since these regions would not be exposed to the release of synaptic vesicle content, this event could mask the changes in pH reported by our SEP-BTX sensor in the synaptic cleft. However, it has been established that extra synaptic acetylcholine receptors are not expressed in normal adult mouse muscle both, by iontophoretic application of acetylcholine^18,19^ and by using labelled bungarotoxin^20^. Moreover, we focused the detection of fluorescent changes at the synaptic spot where only synaptic acetylcholine receptors are located. Secondly, it is worth mentioning that our measurements are highly dependent on the distance of the probe in relation with the source of the protons, which in our case is the synaptic cleft width. The high mobility of protons^21^ and the presence of buffers in the media could result in a very fast transient non accumulative increase in proton concentration not resolved with our detection system. Furthermore, the rapid dissipating proton concentration during the intervals between evoked transmitter release (10 ms at 100 Hz and 3.3 ms at 300 Hz) will strongly down average the fluorescent signal.

In summary, using a novel approach, in contrast to other reports showing an alkalization at CNS synapses^8,22,23^, we were able to show for the first time an acidification process due to transmitter release at the mouse NMJ. Synaptic cleft acidification and alkalization are probably not mutually exclusive processes. While they could operate in different types of synapses, it is also feasible that they occur at the same synapsis but with different kinetics. For example, following a rapid drop in pH, an over compensating mechanism driven by the Ca^2+^/H^+^ antiporter activity of plasma membrane Ca^2+^ ATPase^4^ and aimed at maintaining proton homeostasis could result in transient alkalinization^8^. Further experimental development of sensors located at the presynaptic membrane close to the vesicle release site and a fast image recording system are needed to obtain more realistic measurements of changes in the pH at the synaptic cleft.

## Methods

### Fusion protein

The DNA fragment containing SEP^9^ in frame with BTX and followed by a TEV protease site and a 6XHis tag was subjected to codon optimization for expression in *P. pastoris* and synthetized by Integrated DNA Technologies (IDT). The sequence of the cloned DNA fragment, including *Eco*RI sites, is as follows:

5′GAATTCTCTAAGGGTGAAGAGCTTTTCACCGGCGTGGTTCCTATTCTTGTGGAATTGGATGGTGACGTCAATGGTCACAAATTTTCAGTGTCAGGTGAAGGGGAGGGTGACGCCACTTACGGTAAATTGACTTTGAAGTTTATATGCACTACCGGCAAATTGCCAGTTCCATGGCCTACCTTGGTTACTACCTTAACTTACGGTGTTCAATGCTTTTCTAGATATCCTGACCATATGAAGAGACATGATTTTTTCAAGTCAGCCATGCCAGAGGGATATGTACAAGAACGTACAATTTTTTTTAAGGATGACGGTAATTACAAGACTCGTGCAGAAGTTAAGTTCGAAGGCGACACGCTGGTCAATCGTATTGAACTGAAGGGTATTGACTTCAAGGAGGATGGGAACATACTAGGACACAAATTAGAATATAATTATAACGATCACCAGGTTTACATTATGGCTGACAAACAAAAGAACGGCATTAAAGCTAACTTTAAAATTCGTCATAATATTGAGGATGGGGGAGTCCAATTAGCAGATCATTACCAACAGAATACACCAATAGGAGATGGTCCAGTGCTGTTGCCTGATAATCACTATCTTTTCACTACTTCTACTTTATCTAAAGACCCAAACGAGAAGAGGGACCATATGGTTTTGCTGGAATTTGTGACTGCAGCCGGGATTACTCACGGTATGGACGAGCTTTATAAAGGGCACGTCGGGATAGTTTGCCATACGACAGCCACCTCCCCCATTTCTGCTGTTACCTGTCCTCCAGGCGAAAACCTGTGCTACAGAAAAATGTGGTGTGATGCCTTCTGTTCTTCACGTGGTAAAGTTGTCGAGCTGGGATGCGCTGCCACCTGTCCTAGTAAGAAACCTTATGAAGAAGTAACATGTTGCTCTACAGATAAATGTAATCCACATCCTAAACAACGTCCTGGTTCGCGAGAGAACCTGTACTTTCAAGGACCCGGGCATCATCATCACCATCATTAAATTTAAATGAATTC3′. This fragment was cloned into the *Eco*RI site of pPic9 (Invitrogen Life Technologies Inc.) in frame with *Saccharomyces cerevisiae* α-factor signal sequence.

### Expression system

The protocol for protein expression in *P. pastoris* was previously described^24^. Basically, pPic9-SEP-BTX-TEV-6XHis vector was linearized with *Dra*I and used for transformation of *P. pastoris* strain GS115 (Invitrogen Life Technologies) by electroporation, and recombinant clones reverting histidine auxotrophy were selected on minimal MD plates (0.34% yeast nitrogen base without amino acids, 10 g/L (NH_4_)_2_SO_4_, 2% dextrose and 2% agar). Selection of clones expressing and secreting SEP-BTX was performed by transferring colonies to a nitrocellulose membrane placed on plates of MM minimal medium (0.34% yeast nitrogen base without amino acids, 10 g/L (NH_4_)_2_SO_4_, and 2% agar) with methanol as sole carbon source, for induction of the AOX1 promoter (*P. pastoris* alcohol oxidase 1). After 4 days of growth at 30 °C, the nitrocellulose membranes were washed with distilled water and the secreted protein was revealed by western blot using a polyclonal rabbit anti-HIS antibody (Genescript, USA). For SEP-BTX production, recombinant clones were grown in 50 mL of BMGY medium (1% yeast extract, 2% peptone, 0.34% yeast nitrogen base without amino acids, 10 g/L (NH_4_)_2_SO_4_, 400 mg/L biotin, 1% glycerol, 100 mM potassium phosphate buffer, pH 6.0) for 48 h at 30 °C and 220 rpm. Cells were harvested by centrifugation for 5 min at 1500 g and resuspended in BMMY medium (1% yeast extract, 2% peptone, 100 mM potassium phosphate buffer, pH 6.0, 0.34% yeast nitrogen base without amino acids, 10 g/L (NH_4_)_2_SO_4_, 400 mg/L biotin, 200 µM CuSO_4_ and 3% sorbitol) to a final OD_600 nm_ = 10 and cultivated in 1000 mL shake flasks at 28 °C and 220 rpm. Sterile methanol (0.5% final) was added every 24 h to maintain induction conditions.

### Purification of recombinant SEP-BTX fusion protein and western blot

The protocol for protein purification from *P. pastoris* was previously described^24^. Briefly, *P. pastoris* cultures were harvested after 4 days of induction in BMMY and centrifuged at 1500 g for 10 min. The extracellular supernatant was concentrated by ultrafiltration (30 kDa MWCO, Amicon Ultra, Merck Millipore) and buffer exchanged to 300 mM NaCl, 50 mM sodium phosphate buffer, pH 8, containing 1 mM PMSF. SEP-BTX was purified by gravity flow Ni–NTA affinity chromatography using a “His select” nickel affinity gel (Sigma Chemical Co., USA), eluted with 300 mM NaCl, 50 mM sodium phosphate buffer, pH 6.5, 250 mM imidazole, 1 mM PMSF. Fractions containing SEP-BTX were pooled and buffer exchanged to 1× PBS by ultrafiltration (10 kDa MWCO, Amicon Ultra, Merck Millipore). Recombinant SEP-BTX was revealed by western blot. Culture supernatants and purification fractions were separated by reducing 12% SDS-PAGE and transferred to 0.45 μm nitrocellulose membranes (Bio-Rad Laboratories Inc, USA). Western blot was performed by probing the membranes with 0.1 μg/mL of polyclonal rabbit anti-HIS antibody (Genescript, USA) or a 1:1000 dilution of mouse anti-GFP antibody (Abcam) followed by 1:15,000 dilution of alkaline phosphatase-linked goat anti-rabbit antibody or goat anti-mouse antibody (Sigma Chemical Co., USA). Phosphatase activity was revealed by a chromogenic reaction using 5 bromo-4 chloro-3 indolyl phosphate and nitroblue tetrazolium as substrates (Sigma Chemical Co., USA).

### Animal protocols

Experiments were carried out on the left *Levator auris longus* (LAL) muscle of male C57BL/6 J mice (https://www.jax.org/strain/000664) as previously described^25^. Briefly, animals were supplied by the animal house of the FCEN-UBA. Animals were cared for in accordance with national guidelines for the human treatment of laboratory animals, similar to those of the US National Institutes of Health. All experimental protocols were approved by the Institutional Commission for the Care and Use of Laboratory Animals (CICUAL), FCEN-UBA. Animals were anesthetized with an overdose of 2% tribromoethanol (0.15 mL/10 g body weight) injected in the peritoneal cavity and exsanguinated immediately.

The muscle with its nerve supply was excised and dissected on a Sylgard-coated Petri dish containing physiological saline solution of the following composition (in mM): 137 NaCl, 5 KCl, 2 CaCl_2_, 1 MgSO_4_, 12 NaHCO_3_, 1 Na_2_HPO_4_ and 11 glucose; continuously bubbled with 95% O_2_/5% CO_2_; pH 7.3 as previously described^25^.

LAL muscles were incubated in Ringer´s saline solution with the SEP-BTX sensor for 2 h and washed for 30 min. For each batch of SEP-BTX produced, containing 30–50 ng/µL of purified recombinant protein, preliminary experiments were done to identify the dilution (1/5 to 1/20) necessary to obtain a low background strong labelled NMJ. The preparation was then transferred to a 1.5 mL recording chamber. Experiments were performed at room temperature (20–23ºC). NMJs were visualized with Nomarsky interference optics and illuminated with a 488 nM light source (T.l.L.L Photonics, Germany).

The nerve was stimulated via two platinum electrodes isolated with vacuum grease (Down Corning, USA) coupled to a pulse generator (Grass S88, Grass Inst., USA). Supra threshold high frequency (50, 100 or 300 Hz) nerve stimulation was applied during 250 ms. To avoid using high concentrations of the reporter and to prevent any remaining muscle contraction during stimulation, curare up to 5 × 10^–6^ g/mL (Sigma Aldrich, USA) was used during the experiment.

### Live imaging acquisition and analysis

For fluorescent image acquisition, we used a BX51WI upright microscope with a 60X objective lens (Olympus) and an electron multiplying CCD camera (Andor iXon, Oxford Instruments), together with cell^M System Coordinator/cell^R real time controller software.

The only exception is the image shown in Fig. 1C, for which we used a FV300 confocal fluorescence microscope (Olympus Optical, Tokyo, Japan) with a 60 × objective lens (Olympus), equipped with an image-acquisition system and Fluoview 3.3 software (Olympus Optical). Off-line image processing was performed using ImageJ. First, fluorescence images (Binnig ×2) of 4 ms exposure every 20 ms were acquired before, during and after stimulation and repeated 10 times every 7 s. In each image the average fluorescence signal in a region of the NMJ was “background corrected” by subtraction of the average measured signal from a muscle non NMJ region. The 10 cycles were normalized and averaged. Fluorescence signals before stimulation (frame 1–10) were averaged (Fo) and each record was plotted as Fx − Fo/Fo (ΔF/Fo) over time. Fluorescence imaging experiments were performed in bicarbonate buffer and HEPES buffer extracellular solution.

### Data analysis and statistics

Data analysis was done using Clampfit 10.6 (Molecular Devices, USA), Sigma Plot 10.0, SigmaStat 3.5 and Excel 2007 (Microsoft). Average data are expressed and plotted as mean ± sem. Statistical significance was determined using paired or unpaired Student’s *t*-test.

## Data Availability

All data generated or analyzed during this study are included in this published article.

## References

[1] Miesenböck G, De Angelis DA, Rothman JE (1998). Visualizing secretion and synaptic transmission with pH-sensitive green fluorescent proteins. Nature.

[2] Liu Y, Edwards RH (1997). The role of vesicular transport proteins in synaptic transmission and neural degeneration. Annu. Rev. Neurosci..

[3] Cho S, Von Gersdorff H (2014). Proton-mediated block of Ca^2+^ channels during multivesicular release regulates short-term plasticity at an auditory hair cell synapse. J. Neurosci..

[4] Thomas RC (2009). The plasma membrane calcium ATPase (PMCA) of neurones is electroneutral and exchanges 2 H^+^ for each Ca^2+^ or Ba^2+^ ion extruded. J. Physiol..

[5] Krishtal O, Osipchuk YV, Shelest T, Smirnoff S (1987). Rapid extracellular pH transients related to synaptic transmission in rat hippocampal slices. Brain Res..

[6] Palmer MJ, Vigh J, Von Gersdorff H (2003). Synaptic cleft acidification and modulation of short-term depression by exocytosed protons in retinal bipolar cells. J. Neurosci..

[7] Du J (2014). Protons are a neurotransmitter that regulates synaptic plasticity in the lateral amygdala. Proc. Natl. Acad. Sci..

[8] Stawarski M (2020). Neuronal glutamatergic synaptic clefts alkalinize rather than acidify during neurotransmission. J. Neurosci..

[9] Sankaranarayanan S, De Angelis D, Rothman JE, Ryan TA (2000). The use of pHluorins for optical measurements of presynaptic activity. Biophys. J ..

[10] Thomsen RH, Wilson DF (1983). Effects of 4-aminopyridine and 3, 4-diaminopyridine on transmitter release at the neuromuscular junction. J. Pharmacol. Exp. Ther..

[11] Beckwith-Cohen B, Holzhausen LC, Wang T-M, Rajappa R, Kramer RH (2019). Localizing proton-mediated inhibitory feedback at the retinal horizontal cell-cone synapse with genetically-encoded pH probes. J. Neurosci..

[12] Vincent PF (2018). Clustered Ca^2+^ channels are blocked by synaptic vesicle proton release at mammalian auditory ribbon synapses. Cell Rep..

[13] Hirasawa H, Yamada M, Kaneko A (2012). Acidification of the synaptic cleft of cone photoreceptor terminal controls the amount of transmitter release, thereby forming the receptive field surround in the vertebrate retina. J. Physiol. Sci..

[14] DeVries SH (2001). Exocytosed protons feedback to suppress the Ca^2+^ current in mammalian cone photoreceptors. Neuron.

[15] Wang T-M, Holzhausen LC, Kramer RH (2014). Imaging an optogenetic pH sensor reveals that protons mediate lateral inhibition in the retina. Nat. Neurosci..

[16] González-Inchauspe C, Urbano FJ, Di Guilmi MN, Uchitel OD (2017). Acid-sensing ion channels activated by evoked released protons modulate synaptic transmission at the mouse calyx of held synapse. J. Neurosci..

[17] Urbano FJ (2014). Acid-sensing ion channels 1a (ASIC1a) inhibit neuromuscular transmission in female mice. Am. J. Physiol.-Cell Physiol..

[18] Axelsson J, Thesleff S (1959). A study of supersensitivity in denervated mammalian skeletal muscle. J. Physiol..

[19] Miledi R (1960). Junctional and extra-junctional acetylcholine receptors in skeletal muscle fibres. J. Physiol..

[20] Berg DK, Kelly RB, Sargent PB, Williamson P, Hall ZW (1972). Binding of α-bungarotoxin to acetylcholine receptors in mammalian muscle. Proc. Natl. Acad. Sci..

[21] Cukierman S (2006). Grotthuss! and other unfinished stories. Biochim. Biophys. Acta (BBA) Bioenerg..

[22] Chen H-Y, Chesler M (2015). Autocrine boost of NMDAR current in hippocampal CA1 pyramidal neurons by a PMCA-dependent, perisynaptic, extracellular pH shift. J. Neurosci..

[23] Makani S (2012). NMDA receptor-dependent afterdepolarizations are curtailed by carbonic anhydrase 14: regulation of a short-term postsynaptic potentiation. J. Neurosci..

[24] Campos PA, Levin LN, Wirth SA (2016). Heterologous production, characterization and dye decolorization ability of a novel thermostable laccase isoenzyme from Trametes trogii BAFC 463. Process Biochem..

[25] Bertone NI (2017). Carbonic anhydrase inhibitor acetazolamide shifts synaptic vesicle recycling to a fast mode at the mouse neuromuscular junction. Synapse.

