# Author Correction: A new tool to sense pH changes at the neuromuscular junction synaptic cleft

**DOI:** 10.1038/s41598-021-88219-2

**Published:** 2021-04-15

**Authors:** Matías Blaustein, Sonia Wirth, Gustavo Saldaña, Ana Paula Piantanida, María Eugenia Bogetti, María Eugenia Martin, Alejandro Colman-Lerner, Osvaldo D. Uchitel

**Affiliations:** 1 Departamento de Fisiología, Biología Molecular y Celular, Facultad de Ciencias Exactas y Naturales (FCEN), Universidad de Buenos Aires (UBA), Buenos Aires, Argentina; 2 Instituto de Biociencias, Biotecnología y Biología Traslacional (iB3), FCEN, UBA, C1428EHA Buenos Aires, Argentina; 3 Instituto de Biodiversidad y Biología Experimental y Aplicada (IBBEA), FCEN, CONICET-UBA, Buenos Aires, Argentina; 4 Instituto de Fisiología, Biología Molecular y Neurociencias (IFIBYNE), CONICET-UBA, C1428EHA Buenos Aires, Argentina; 5 Instituto de Biología Celular y Neurociencias (IBCN) Dr. Eduardo de Robertis, Facultad de Medicina, CONICET-UBA, Buenos Aires, Argentina

Correction to: *Scientific Reports* https://doi.org/10.1038/s41598-020-77154-3, published online 24 November 2020

The original version of this Article contained errors in the spelling of the authors Matías Blaustein, Sonia Wirth, Gustavo Saldaña, Ana Paula Piantanida, María Eugenia Bogetti, María Eugenia Martin, Alejandro Colman-Lerner, Osvaldo D. Uchitel which were incorrectly given as M. Blaustein, S. Wirth, G. Saldaña, A. P. Piantanida, M. E. Bogetti, M. E. Martin, A. Colman-Lerner, O. D. Uchitel respectively.

These errors have now been corrected in the PDF and HTML versions of the Article.

